# The development of blended friendship in high leader-member exchange relationships: Mechanisms and consequences of a relational shift

**DOI:** 10.1177/20413866241269739

**Published:** 2024-08-09

**Authors:** Marie-Colombe Afota, Ariane Ollier-Malaterre, Christian Vandenberghe

**Affiliations:** 141639 Université de Montréal, Canada; 14845 Université du Québec à Montréal, Canada; 10014 HEC Montréal, Canada

**Keywords:** leader-member exchange, blended friendship, multiplex relationships, inter-role enrichment, inter-role conflicts

## Abstract

Confusion persists about the overlap between high-quality leader-member exchange (LMX) relationships and personal friendships between a leader and a subordinate. How these notions differ, shift from one to the other, and what their consequences are remain unclear. This paper proposes a framework that examines the fundamental differences between high LMX relationships and friendships. We argue that when high LMX relationships shift toward friendships, they in fact shift toward *blended friendships*, where the leader and the subordinate concomitantly enact two distinct roles, worker and friend. These blended friendships are qualitatively different from high LMX and from friendships. We detail the process by which blended friendship develops in the context of high LMX relationships and identify the key variables and mechanisms that drive the emergence of such blended friendships. We then examine how subordinates’ well-being, job engagement, performance, and turnover may simultaneously benefit and suffer from their involvement in a blended friendship.

## Plain Language Summary

For decades, the leadership literature has emphasized the importance of leader–subordinate relationships, providing abundant evidence that high-quality relationships between leaders and subordinates lead to a host of positive outcomes. Yet, significant confusion persists about the configurations of high-quality leader-subordinate relationships. How such relationships overlap and differ from personal friendships between leaders and subordinates, how they may morph into friendships, and what the outcomes of such relational change are remain unclear. This paper offers a theoretical framework that brings clarity to these issues. We examine the fundamental differences between high quality leader-subordinate relationships and friendships. We argue that when leaders and subordinates involved in a high-quality relationship get closer, their relationship may not turn into a friendship, but into a *blended friendship*, in which each partner must concomitantly behave as a friend and as a leader/subordinate. We examine how such relational shift may occur and identify key precipitating factors. We then explain how a blended friendship between a leader and a subordinate may lead to both positive and negative outcomes due to either enrichment or conflict between the friend's and the worker's role. The proposed framework clarifies the position of leaders and subordinates within their relationships with reference to friendship and sheds light on the dynamic nature of these relationships.

**“**Should you be friend with your boss?” asked an article in *Forbes* (Wuench, 2021), while the *Wall Street Journal* pointed out “the potentially high cost of being friends with the boss” (Mitchell, 2019). Friendship between leaders and subordinates and its associated risks are frequently discussed in the popular press. Yet, the academic literature is not clear about the outcomes of such relationships. Relationships between leaders and subordinates are predominantly examined through the leader-member exchange (LMX) lens (Graen & Scandura, 1987; Graen & Uhl-Bien, 1995), which posits that leaders and subordinates develop relationships of varying quality, with high-quality LMX relationships yielding the most favorable consequences (Dulebohn et al., 2012). High-quality LMX relationships are characterized by mutual trust, support, liking, and loyalty (Graen & Uhl-Bien, 1995). These characteristics resemble those of friendship (Morrison & Cooper-Thomas, 2016), which reflects the affective dyadic bond (Hays, 1998) that belongs to the private sphere (Rawlins et al., 1986). However, how high-quality LMX may come to incorporate friendship and the consequences of such a shift remain unclear (Horan et al., 2021). A better understanding of why, how, and with what consequences high LMX may shift to potentially less functional relationships is critical because abundant evidence points to high-quality LMX as the holy grail of leadership effectiveness (DeRue & Myers, 2014).

The first objective of this paper is to bring clarification into the conceptual distinctions between high LMX and friendships. Such a clarification is needed because the presence of affect/liking as a key feature of high LMX relationships has created persistent confusion (Dulebohn et al., 2017): some scholars have suggested that high LMX involves friendship (e.g., Maslyn & Uhl-Bien, 2001) while others make a sharp difference between work and nonwork ties (e.g., Graen & Uhl-Bien, 1995; Nifadkar et al., 2019), arguing that LMX and friendship are distinct relationships.

Our second objective is to explain how and under what conditions high LMX relationships shift over time and incorporate friendships’ characteristics. Although the LMX literature has mostly adopted a static orientation, relationships change (Gibson, 2018) and LMX relationships are no exception (Cropanzano et al., 2017; Ellis et al., 2019; Park et al., 2015). To our knowledge, Boyd and Taylor's depiction (1998) of the development of LMX and friendship ties as parallel and concurrent processes is the only work of this nature. Our perspective differs from that work by focusing on the development of friendship within the context of established high LMX. Work relationships (e.g., LMX relationships) are likely to precede friendships because relational partners need time to get to know each other before accepting the risks inherent to intimacy (Gibson, 2018). Indeed, LMX relationships form quickly (Nahrgang & Seo, 2015), while friendships necessitate more time to develop (Derlega et al., 2008).

Our third objective is to examine the mechanisms and outcomes of friendship bonds between leaders and subordinates. Although the workplace relationships literature has begun to examine workplace friendships (e.g., Methot et al., 2016; Pillemer & Rothbard, 2018) and identified specific relational risks (e.g., favoritism, manipulation; Boyd & Taylor, 1998; Bridge & Baxter, 1992), subordinates’ responses to these tensions and the full spectrum of the potential risks and benefits of forming a close relationship with a leader are still to be explored.

To address the above gaps, we reexamine the LMX literature through the lens of relationship science and argue that, though closely related, high LMX relationships and friendships are qualitatively distinct and to some extent discordant. We specifically contend that some high LMX relationships may shift towards blended friendships, i.e., multiplex dyadic relationships that involve the simultaneous enactment of a friend role and a work role (Bridge & Baxter, 1992; Haythornthwaite, 2001) that are worthy of attention. Building upon the relationship development literature (Altman et al., 1981; Derlega et al., 2008; Gibson, 2018), we identify the key variables that drive such a shift and develop a processual model of the events that push high LMX relationships toward blended friendships. Finally, we examine how subordinates involved in a blended friendship with their leader may simultaneously experience inter-role enrichment and conflicts, leading to both positive and negative outcomes. For the sake of parsimony, we focus on a limited set of outcomes that pertain to the triad of psychological health (emotional exhaustion and job engagement), behaviors (performance), and attitudes (turnover intention). Emotional exhaustion is a crucial indicator of psychological health (Maslach & Leiter, 2008) while performance and turnover intention are among the most established consequences of high LMX (Dulebohn et al., 2012).

The scope of our work is framed by three contextual boundaries (the “when/where/who” of the theory; Whetten, 1989). First, we focus on high LMX relationships rather than the full range of LMX relationships because low LMX involves low liking and trust and is therefore unlikely to shift into blended friendships. Second, though friendship formation is a dyadic process that involves the leader and the subordinate, we restrain our theorizing to *subordinates’* perceptions and experiences. Dyadic partners’ perceptions of their work relationships—including LMX—are indeed only moderately correlated (Byron & Landis, 2020) and blended friendships may generate different challenges for leaders and subordinates. Third, while we acknowledge that friendship might, in some situations, precede LMX relationships (e.g., after an internal promotion within the team; see Unsworth et al., 2018 for a discussion on how leaders experience such situations), our focus is on the more usual circumstances where the work relationship precedes the personal relationship.

**Figure 1.**
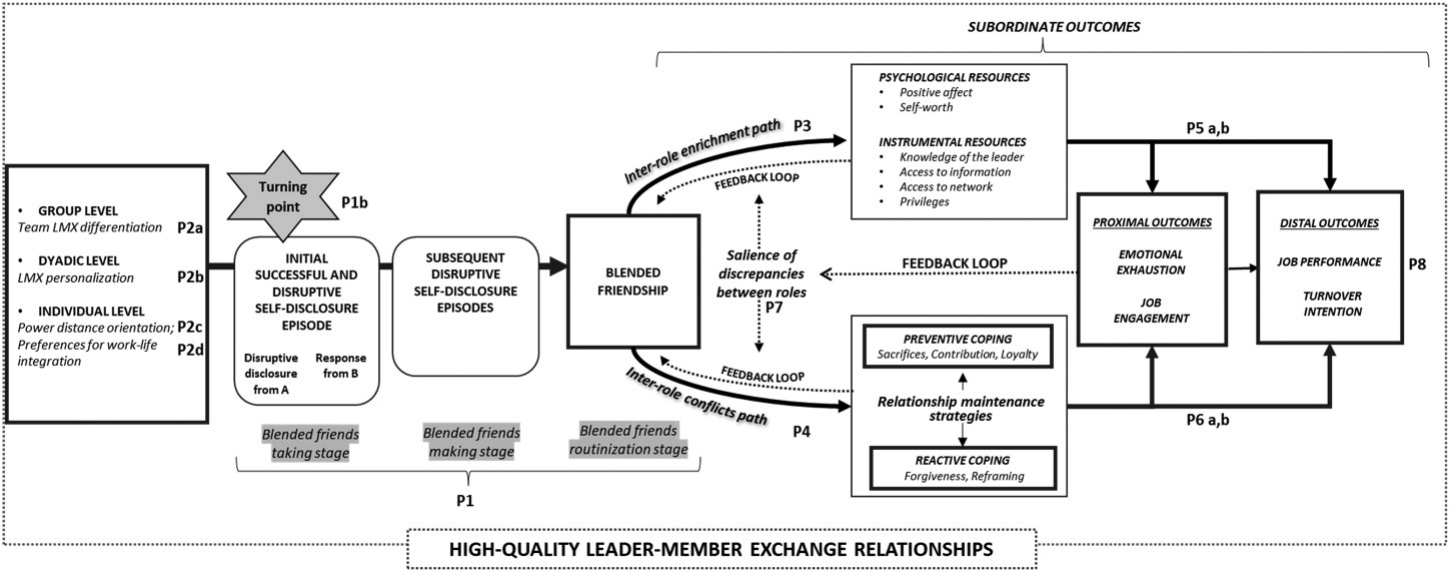
A longitudinal perspective illustrating how high LMX may lead to blended friendship over time and its consequences. LMX, leader-member exchange.

Our proposed framework (Figure 1) makes several contributions. First, we contribute to the LMX literature by clarifying the uniqueness of the LMX construct compared to friendships and blended friendships, two types of relationships that we argue resemble but are distinct from high LMX. This is a much-needed endeavor because the formation of personal and multiplex ties is frequent in the workplace (Song et al., 2020) and may therefore occur in high LMX relationships. Thus, failing to differentiate high LMX relationships from friendships and blended friendships may limit scholars’ ability to grasp the full meaning of the relationships they observe. Second, we explore the drivers and processes by which a high LMX relationship may turn into a blended friendship. Until very recently, the literature has assumed that LMX relationships tend to remain stable, hence focused on LMX levels across people rather than on within-person change in LMX, thereby neglecting the fact that interpersonal relationships are dynamic (Dimotakis et al., 2023). Recent research examined short-term changes in LMX (e.g., Dimotakis et al., 2023; Ellis et al., 2019) and reported evidence for change in LMX over long periods of time (e.g., Epitropaki et al., 2020). Complementing these findings that centered on the fluctuations in LMX *levels*, we offer a depiction of the mechanisms that underlie shifts in LMX *content*. Third, our model contributes to the literature on multiplex ties (Bridge & Baxter, 1992; Haythornthwaite, 2001; Methot et al., 2016) by presenting a nuanced understanding of subordinates’ responses to a blended friendship with the leader. Rather than examining the consequences of high LMX and friendship separately (e.g., Ramarajan et al., 2017), we examine both the bright (i.e., role enhancement) and dark (i.e., role conflicts) sides of role co-activation (Ramarajan et al., 2017) in the context of leader-subordinate blended friendships.

## High-quality LMX, friendship, and blended friendship

**Figure 2.**
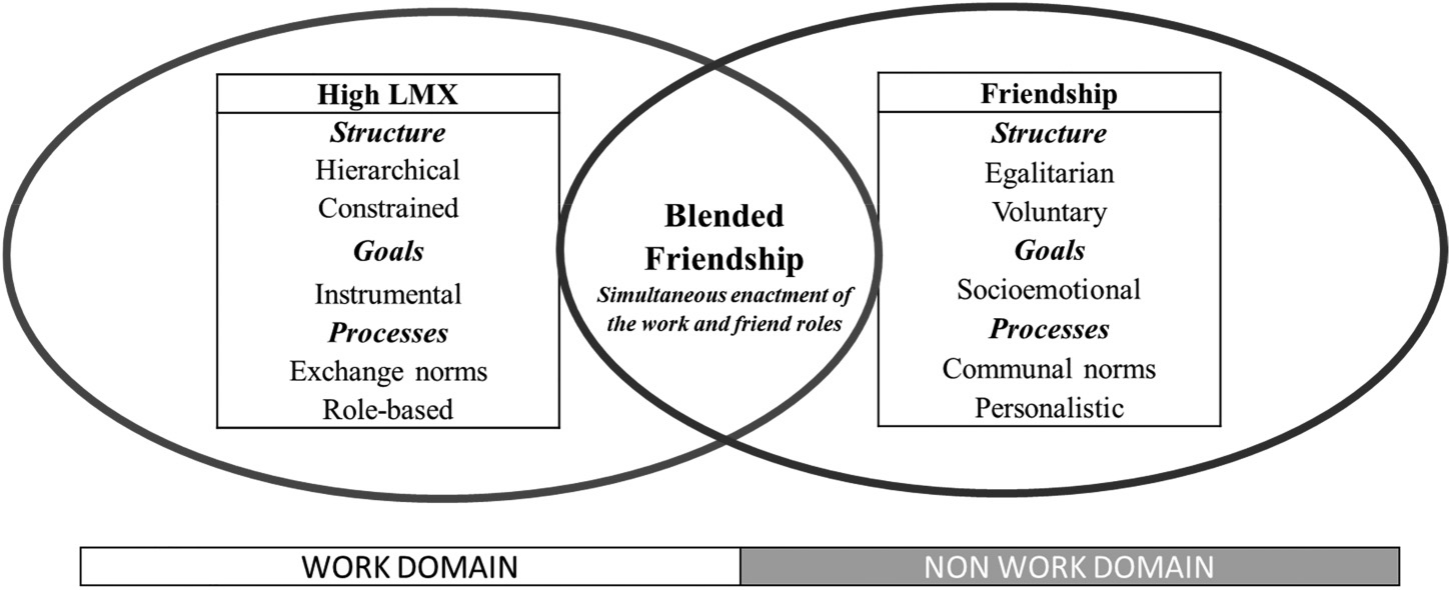
High LMX, friendship, and leader-subordinate blended friendship: commonalities and differences. LMX, leader-member exchange.

We begin by clarifying the differences among high LMX, friendship, and blended friendship. Figure 2 summarizes them, based on Koerner's relationship typology (2018) along with their structure, goals, and processes.

### LMX relationships

The structure of LMX is one of *power asymmetry*, even if reduced in high LMX relationships (Boyd & Taylor, 1998), and of *exogeneity* since the relationship is constrained by the rules of the work environment. The goals of LMX are primarily *instrumental* (Barry & Crant, 2000) because work relationships are inherently “directed at the accomplishment of some common objectives or goals” (Ferris et al., 2009, p. 1379). These are governed by a process of *social exchange* (Blau, 1964) rather than by communal ties where partners are driven by a desire to support each other's well-being, without expecting reciprocity (Clark & Mils, 1993). Finally, LMX involves *role-based* relationships that develop during a role negotiation process (Graen & Scandura, 1987) rather than through personalistic interactions where individuals take precedence over the roles (Sias & Cahill, 1998).

### Friendships

Friendships are mutual and voluntary relationships (Hays, 1998) that “exist primarily for personal satisfaction and enjoyment rather than for the fulfillment of a particular task or goal” (Sapadin, 1988, p. 387) and in which “the participants respond to one another personally, that is, as unique individuals rather than as packages of discrete attributes or mere role occupants” (Wright, 1984, p. 119). Friendships are generally viewed as belonging to the non-work domain (Bridge & Baxter, 1992) because their voluntary and non-instrumental nature as well as their focus on the whole person (Horan et al., 2021) are misaligned with the instrumental role-based relationships of the work domain (Pillemer & Rothbard, 2018; Styhre, 2000). Yet, the workplace is fertile ground for the development of friendships (Sias & Cahill, 1998). Friendships are typically *egalitarian* and *voluntary* (Sias & Cahill, 1998), serve *socioemotional* goals (Pillemer & Rothbard, 2018), and are *communal* (Clark & Mils, 1993) and *personalistic*, involving interactions between unique and irreplaceable individuals (Sias & Cahill, 1998).

While high LMX relationships and friendships share liking, trust, and positive affect, they differ qualitatively as their key feature expressions are, to a large extent, discordant (Bridge & Baxter, 1992; Horan et al., 2021; Pillemer & Rothbard, 2018). For example, regardless of how close the leader and subordinate may be, the power asymmetry between them persists such that true equality can never be reached. Thus, though high LMX members may become more intimate and develop friendship-like relationships (Styhre, 2000), we argue that such relationships should not be viewed as friendships per se, but rather as *blended* friendships (Ashforth & Sluss, 2006; Bridge & Baxter, 1992; Haythornthwaite, 2001; Methot et al., 2016).

### Leader-subordinate blended friendships

Blended friendships, also known as multiplex friendships (Methot et al., 2016), combine the work and personal domains (Bridge & Baxter, 1992). In such relationships, individuals simultaneously enact two roles - the worker role and the friend role. Of course, friendships are not altogether absent from work relationships. For instance, one item of the Multidimensional Measure of LMX (LMX-MDM; Liden & Maslyn, 1998) reads: “My supervisor is the kind of person one would like to have as a friend.” However, friendships that develop between leaders and subordinates in formal work roles inevitably incorporate work-based role expectations, hence becoming blended rather than mere friendships. Blended friendships differ from high LMX relationships and friendships by *blurring* the line between work and non-work domains (Horan et al., 2021) and *simultaneously* enacting the work and friend roles. In a leader-subordinate blended friendship, subordinates must abide by both the role expectations of subordinate (someone with less formal power) and friend (equal-status). Such co-activation paves the way for inter-role conflicts (Ashforth & Sluss, 2006; Bridge & Baxter, 1992; Dumas et al., 2013) as well as inter-role enrichment (Greenhaus & Powell, 2006; Ramarajan et al., 2017).

## The development of blended friendship

### Disruptive and successful self-disclosure as a turning point

The literature on workplace relationships increasingly acknowledges their dynamic nature (Gibson, 2018; Schinoff et al., 2020). Although high LMX relationships have been considered relatively stable once established (Graen & Uhl-Bien, 1995; Nahrgang & Seo, 2015), routinized LMX relationships may change (e.g., Park et al., 2015), particularly after a critical incident (Cropanzano et al., 2017). For a qualitative change to occur in a relationship, there must be an initial disruptive or anchoring episode (Ballinger & Rockmann, 2010) that acts as a turning point. Gibson's theory of relational change (2018) suggests that the initial turning point toward a closer bond generally takes the form of a disruptive self-disclosure, in which a discloser shares “previously unknown self-relevant information that challenges current expectations” (Gibson, 2018, p. 571).

Self-disclosures, which are verbal (e.g., revealing a mental health condition, inviting to meet outside of work) or nonverbal (e.g., a friend request on social media; Rothbard et al., 2022) sharing of personal information, thoughts, or feelings, nurture friendship development (Altman & Taylor, 1973; Collins & Miller, 1994) because they serve as signals communicating the discloser's desire for intimacy (Collins & Miller, 1994). In the workplace, self-disclosures are disruptive if they significantly deviate from routinized role expectations, prompting the relational partner to reassess the relationship's significance (Gibson, 2018; Reis & Shaver, 1988). Typically, sharing nonwork information is disruptive as it is unexpected in organizational contexts (Nifadkar et al., 2019).

High LMX relationships are prone to disruptive self-disclosure episodes because trust, liking, and frequent interactions are powerful triggers of self-disclosure (Collins & Miller, 1994). Individuals are more likely to self-disclose to someone they like and trust because self-disclosing is risky (Collins & Miller, 1994). Importantly, a disruptive self-disclosure eventually shifts the relationship's nature towards increased closeness if the receiver appraises the disclosure as goal-congruent, i.e., aligned with their desires for the future of the relationship. In such cases, the receiver is likely to express consent to move toward a closer relationship by demonstrating enthusiasm, empathy, or active listening (Reis & Shaver, 1988). In a high LMX relationship, where mutual liking, care, and concern already exist, self-disclosure by partner A is likely to be assessed by partner B as congruent with their goals, thus eliciting a positive response (Gibson, 2018). For example, a Facebook friend's request from the supervisor would normally lead the subordinate to pause because it deviates from the expectations associated with the negotiated role (Rothbard et al., 2022). However, in a high LMX relationship, such deviation may be interpreted as an expression of appreciation and a willingness to develop a closer bond (Collins & Miller, 1994; Phillips et al., 2009), which remains congruent with the existing bond between the partners.

Notably, what constitutes a disruptive self-disclosure depends on the roles that have been negotiated during LMX formation (Graen & Scandura, 1987). LMX relationships develop through a role-making process in which each partner negotiates their role (Graen & Scandura, 1987) such that certain behaviors are consistent with these roles (e.g., inquiring about one's plans for the weekend), while others are not (e.g., sending a nonwork-related text during the weekend). Thus, a disruptive self-disclosure can take multiple forms and its disruptive intensity may vary depending on how partners have defined their role during LMX development.

### Stages of blended friendship

We propose that a successful initial disruptive self-disclosure episode—where one member reveals personal information and the other responds positively—acts as the *blended friendship-taking stage* of the evolving relationship. In line with the cyclical models of relational development (Derlega et al., 2008; Reis & Shaver, 1988), if the first disruptive self-disclosure episode is successful, then a series of subsequent episodes—*the blended friendship-making stage*—will lead both the leader and the subordinate to regularly transgress their respective work roles, alternately self-disclose (Greene et al., 2006), discuss a broad variety of topics deeply (Derlega et al., 2008) and span work and nonwork boundaries (Nifadkar et al., 2019). As partners become more intimate and learn more about facets of each other, their role relationship is likely to become more personal, serve socioemotional goals, and abide by communal instead of exchange norms (Ballinger et al., 2010). However, the markers of the work role relationship remain. The relationship will thus shift toward a blended friendship (*blended friendship routinization stage*), not a friendship.

The shift in the relational trajectory may vary in speed. Theoretical and empirical support exists for both the slow and gradual development predicted by social penetration theory (Altman & Taylor, 1973) and the rapid and sudden shift predicted by the clicking model (Berg & Clark, 1986). Indeed, disruptive self-disclosures vary in intensity (Gibson, 2018), such that low-intensity disclosures (e.g., telling a joke) engender slower patterns of relational development than high-intensity disclosures (e.g., an invitation to a birthday party).

Moreover, the friendship formation process is neither necessarily linear nor automatically successful (Perlman et al., 2015). For example, Johnson et al. (2003) found evidence for five trajectories of relationship development, some of which included momentary downturns before eventually progressing. Partners may also pause their journey towards greater closeness or momentarily move away from each other due to situational constraints or after an episode (e.g., minor conflict) that prompts them to reevaluate the relationship (Johnson et al., 2003). The blended friendship development formation process between high LMX partners may also start but then come to a halt. Such a situation can occur if a partner discloses information that the other perceives as inappropriate or too disruptive (e.g., sharing too much too soon), which may lead the target of the self-disclosure to react negatively (e.g., acts like nothing had been said). Such an eschewal response will likely put an end to relationship development (Fehr, 2008). The blended friendship formation process may also be unsuccessful if one partner expresses a lack of genuine interest in building a closer bond (Derlega et al., 2008). This can occur if a subordinate interprets the leader's self-disclosure as being motivated by instrumental goals (e.g., intentionally faking closeness to serve work team performance) or as being impersonal, for instance when the subordinate realizes that the leader has similarly self-disclosed to others (Derlega et al., 2008).

**P1a.** Over time, high LMX relationships may transition into blended friendships. This shift results from an initial disruptive and successful self-disclosure episode followed by a series of role deviation episodes during which the leader and the subordinate depart from their respective work roles.

Exiting one's role comes with the risk of eliciting a negative response and being rejected (Greene et al., 2006). In an asymmetrical relationship, the literature suggests that the higher-status partner will be the first to exit their role, as such exiting is less risky for them (Derlega et al., 2008; Phillips et al., 2009). Due to their lower status and dependency on the leader, subordinates may be more reluctant to initiate a disruptive self-disclose (Morrison & Cooper-Thomas, 2016).

**P1b.** The first disruptive self-disclosure episode is more likely to be initiated by the leader than by the subordinate.

## Triggers of successful self-disclosure episodes

Research on personal relationships has identified various drivers of self-disruptive episodes. Some of them (e.g., frequency of interactions, proximity, interpersonal attraction, perceived similarity; Derlega et al., 2008; Greene et al., 2006) overlap with high LMX antecedents; while high LMX may vary on these factors, we expect these variations to remain small. Other drivers (e.g., tendency to open up easily; secure attachment style; Fehr, 2008) have been identified in the literature and do not need further discussion here. We instead focus on four triggers that are likely to be particularly important in high LMX situations. One of these factors—*team LMX differentiation*—pertains to the team level, one— *personalization of the LMX relationship*— reflects differences at the dyadic level, and two others—*power distance orientation* and *preferences for work-life segmentation vs. integration*—reflect individual differences.

### Team level: LMX differentiation

Just as leaders lack sufficient resources to build high LMX relationships with all subordinates (Schyns et al., 2010), individuals develop personal friendships with only a limited number of partners (Fehr, 2008). In other words, a friendship can only develop if partners are available for a new friendship (Fehr, 2008). As the number of alternative partners increases, the likelihood of developing a friendship decreases. Thus, a friendship among high LMX partners is more likely to develop when the leader has limited alternative partners. Most often, leaders have multiple subordinates. We can expect that a friendship is more likely to develop with subordinates with whom the leader has the highest-quality LMX.

Team LMX differentiation is the magnitude of the distribution of LMX scores within the team (Henderson et al., 2008), such that high LMX differentiation is characterized by a broad range of LMX quality within the team. When team LMX differentiation is high, the standing of a subordinate with a high LMX will likely be more salient than when team LMX differentiation is low, because there will be fewer options for forming a friendship. Thus, high team LMX differentiation may increase the likelihood that a personal relationship emerges between LMX partners.

**P2a.** In high LMX contexts, high team LMX differentiation is positively associated with successful disruptive self-disclosure episodes and the development of a blended friendship.

### Dyadic level: personalization

Personalization is the extent to which the supervisor-subordinate relationship is based on personal rather than role-based characteristics (Sluss & Ashforth, 2007). Person-based identities represent the salience of members’ individual traits when they enact their roles (e.g., kind, honest, smart) while role-based identities reflect the roles in the dyad, independent of who enacts them (e.g., a supervisor offers support and assigns tasks; a subordinate reports progress on tasks). LMX relationships incorporate both person- and role-based identities (Sluss & Ashforth, 2007) and role expectations vary from dyad to dyad (Graen & Scandura, 1987). Some leaders and subordinates may primarily hold person-based expectations (i.e., affective and socio-emotional aspects of LMX: “I expect my supervisor/subordinate to be kind”), while others may primarily develop role-based expectations (i.e., instrumental aspects of LMX: “I expect my supervisor/subordinate to offer regular feedback”).

A more personalized high LMX relationship is more likely to morph into a blended friendship for two reasons. First, such a relationship may foster self-disclosure episodes because the leader and subordinate are considered as persons, which is one step closer to being seen as whole persons involved in work as well as other life roles (Sias & Cahill, 1998) than when the leader and the subordinate are considered solely as enacting work roles. Second, a personalized relationship involves higher levels of liking (Sluss & Ashforth, 2007), fostering successful self-disclosure episodes (Greene et al., 2006) where members are more likely to be willing to self-disclose and to react positively to a disruptive self-disclosure.

**P2b.** In high LMX contexts, high LMX personalization is positively associated with successful disruptive self-disclosure episodes and the development of a blended friendship.

### Individual level: power distance orientation and preferences for work-life segmentation vs. integration

Power distance orientation is an individual's tendency to accept and value inequalities in organizational status (Daniels & Greguras, 2014). Power distance orientation encourages status differentiation (Anand et al., 2018), with leaders being more directive and using top-down communication strategies (Brockner et al., 2001) and employees expecting their leader to use authority and showing deference (Daniels & Greguras, 2014). Disruptive self-disclosures between leaders and subordinates are incongruent with power distance (Boyd & Taylor, 1998) because leaders may fear losing power and subordinates may not feel entitled to discuss personal issues with their leaders. Furthermore, leaders and subordinates with high power distance orientation will likely react negatively to a disruptive self-disclosure because such disclosure contravenes their expectations for distance (Gibson, 2018; Wang et al., 2021). To be clear, when both partners are high on power distance orientation, although the threshold for a self-disclosure to be disruptive will be lowered (i.e., sharing a “minor” personal information may be perceived as disruptive), such disruptiveness will likely be perceived as inappropriate because it goes against a propensity to maintain distance with the partner (Gibson, 2018).

**P2c.** In high LMX contexts, high power distance orientation is negatively associated with successful disruptive self-disclosure episodes and the development of a blended friendship.

Moreover, individuals vary in the extent to which they prefer to segment or integrate their work and nonwork lives (Kreiner, 2006; Rothbard & Ollier-Malaterre, 2016). Segmentors prefer to establish clear boundaries while integrators prefer to blur their work and nonwork lives. Integrators are more likely to develop blended friendships for two reasons: first, they may be more willing to share personal information with coworkers; second, they may create more opportunities for self-disclosure by socializing with coworkers outside of work. For example, a supervisor who prefers integrating their work and nonwork lives may organize a teambuilding activity at their home, thus creating a favorable context for self-disclosures. Conversely, segmentors may be less likely not only to engage in self-disclosure but also to react positively to a partner's disruptive self-disclosure as it is misaligned with their preferences (Gibson, 2018).

**P2d.** In high LMX contexts, preference for work-life segmentation is negatively associated with successful disruptive self-disclosure episodes and the development of a blended friendship.

## Inter-role enrichment and conflicts, and subordinate outcomes

The next part of our model examines the outcomes of blended friendships. We argue that two pathways—inter-role enrichment and inter-role conflicts—contribute to improving vs. worsening subordinates’ outcomes, respectively. As per role enrichment theory (Greenhaus & Powell, 2006), participation in a role within one domain may benefit one's performance in a role within another domain because involvement in a role generates various resources (e.g., skills, flexibility, positive affect) that can be successfully invested in other roles through an inter-role enrichment process. Conversely, the inter-role conflict perspective suggests that participating in one role diminishes one's ability to meet requirements in another role.

### Inter-role enrichment and resource gains

Multiplex work relationships have been found to be positively associated with commitment, satisfaction, performance, and positive affect (Methot et al., 2016; Morrison & Cooper-Thomas, 2016). In line with role enrichment theory (Greenhaus & Powell, 2006), we argue that such enrichment is likely to occur in a leader-subordinate blended friendship because more resources are acquired in one role and transferred to the other role.

The literature suggests that being intimate with the leader may provide the subordinate with additional psychological and instrumental resources. In terms of psychological resources, friendship ties produce positive affect because they foster pleasant interactions, feelings of acceptance, safety, and warmth (Sias & Gallagher, 2009). Additionally, befriending a higher-status person (i.e., the leader) may enhance subordinates’ self-worth, as individuals gain status and self-esteem when they are affiliated with high-status others (Cialdini & Richardson, 1980).

In terms of instrumental resources, privileged access to the leader provides subordinates with greater knowledge of the leader's way of thinking and expectations. Frequent and close interactions may also push the leader to share more detailed or confidential information that other team members are not aware of (Morrison & Nolan, 2007). Subordinates may also benefit from the intimate bond with the leader in the form of facilitated access to an extended network. Finally, the friend-subordinate may obtain privileges (e.g., exemption from specific work requirements).

**P3.** Subordinates involved in a blended friendship with their leader will gain additional (a) psychological and (b) instrumental resources.

### Inter-role conflicts and coping strategies

Despite the potential benefits of blended relationships, their dark sides have caught growing attention in the literature (e.g., Bridge & Baxter, 1992; Morrison & Nolan, 2007; Pillemer & Rothbard, 2018). The literature suggests that inter-role conflicts in a leader-subordinate blended friendship derive from subordinates’ expectations for (a) preferential treatment that contravenes workplace fairness and equity norms; (b) equality that deviates from the leader's role to assign tasks, evaluate performance, and reward or punish; and (c) openness that are at odds with asymmetrical access to information and confidentiality (Boyd & Taylor, 1998; Bridge & Baxter, 1992; Unsworth et al., 2018).

To handle these role incompatibilities (e.g., Hall, 1972; Unsworth et al., 2018; Van de Vliert, 1981), subordinates sequentially use three approaches: prioritizing one role over another (*role choice*), aligning role expectations (*role compromising*), and, as a last resort, withdrawing from one or both roles (*role elimination*). In a blended friendship, prioritizing one role is not feasible because both roles are tied to the same relational partner. Subordinates are more likely to compromise and seek to align their subordinate and friend roles to protect the relationship (Epitropaki et al., 2020; Rusbult, 1980). To do that, we argue that subordinates will use *preventive* and *reactive coping* strategies (Lazarus & Folkman, 1984; Thompson et al., 2007).

Subordinates may employ preventing coping strategies to avoid making contradictions between their roles as friends and workers apparent. We suggest that preventive coping involves three main tactics: making *sacrifices*, maintaining high levels of *contribution*, and demonstrating *loyalty* to the leader. First, subordinates may lower their expectations and/or align them with their leaders’ even if doing so entails making sacrifices. For example, a subordinate in need of a personal leave may refrain from requesting it to avoid eliciting conflict between friendship preferential treatment and workplace equity. Second, subordinates may maintain their work contribution to avoid negative evaluation by the leader, a situation that emphasizes power asymmetry. Third, subordinates may demonstrate loyalty to the leader, for example by publicly supporting the leader's ideas. Such loyalty may prevent situations where the leader-friend would have to emphasize their higher hierarchical status.

Reactive coping aims at limiting the negative consequences of a conflict that has occurred. Research on leaders’ transgressions (e.g., offenses or betrayals) suggests that reactive coping strategies often include two tactics: *forgiving* and *reframing* the situation to minimize the leader's responsibility (Epitropaki et al., 2020). For example, a subordinate may react to a leader's negative feedback by forgiving the leader's transgression or reframing it in a way that reduces the importance of the breach or attributes it to the work context (Epitropaki et al., 2020).

**P4.** Subordinates involved in a blended friendship with their leader will seek to cope with inter-role conflicts by using two types of strategies: preventive strategies that involve making sacrifices, maintaining high contribution, and demonstrating loyalty to the leader, and reactive strategies that involve forgiving and reframing.

### Foreseeable outcomes for subordinates

As per enrichment theory, the accumulation of resources generated by subordinates’ involvement in a blended friendship can enhance the experience associated with the worker's role (Greenhaus & Powell, 2006). Conversely, the relationship maintenance strategies used to deal with inter-role conflicts may be resource-consuming. We thus expect these two processes to act as concurrent countervailing forces on subordinate work outcomes: while the inter-role enrichment path should promote positive outcomes (i.e., lower exhaustion, higher engagement, increased performance, and lower turnover), the inter-role conflict path is expected to have the opposite effects (i.e., higher exhaustion, lower engagement, decreased performance, and increased turnover). In support of this view, Ramarajan et al. (2017) found opposite effects for identity enrichment and conflicts on role immersion, a proxy of engagement.

#### Proximal outcomes: emotional exhaustion and job engagement

The job demands-resources (JD-R) model (Demerouti et al., 2001) is one of the most prominent frameworks that explain the mechanisms leading to exhaustion and engagement and its focus on demands and resources sheds useful light on the outcomes of blended friendships. This model suggests that job demands cause exhaustion through a resource-depleting process while job resources promote engagement through a resource-accumulation process. Meta-analytic studies have provided strong support for these propositions (e.g., Crawford et al., 2010). The JD-R model further suggests that demands attenuate the relationship between resources and engagement and that resources buffer the relationship between demands and exhaustion. It is worth noting that though exhaustion and engagement have been described as opposites (e.g., Schaufeli et al., 2002), growing evidence has recently accumulated indicating that, while the constructs are negatively related, they are theoretically distinct and worthy of dedicated attention (Byrne et al., 2016). Following these lines, resource gains produced by inter-role enrichment should increase job engagement and reduce subordinates’ emotional exhaustion. Conversely, the strategies that subordinates use to cope with inter-role conflicts can be considered as job demands that likely increase exhaustion and decrease engagement. For example, subordinates who maintain strong work contributions must accept extra work to meet expectations. Those who refrain from claiming what they feel they are entitled to in their friend role, swallow their disagreement or minimize the leader's breaches are likely to cultivate ambivalent feelings toward the leader-friend (Epitropaki et al., 2020). Psychological resources are needed to address the resulting discomfort, as ambivalence violates individuals’ inclinations for consistency (Festinger, 1957).

#### Distal outcomes: performance and turnover intention

Meta-analytic evidence has shown that exhaustion and engagement are (opposite) antecedents to performance and turnover (e.g., Halbesleben, 2010; Lee & Ashforth, 1996). We thus expect resource gains and inter-role conflicts to have contrasting impacts on performance and turnover through their effects on exhaustion and engagement.

In addition to these indirect effects, we also expect direct influences of both resource gains and inter-role conflicts on both performance and turnover. On the positive side, the resource gains are likely to enhance subordinate performance via a deeper knowledge of the leader's functioning, access to an extended network and to a greater amount of information, along with personal resources such as optimism, self-efficacy, and positive emotions. The resources obtained from the blended friendship with the leader will also fuel positive feelings and work satisfaction, which, combined with the attachment to the leader-friend, will result in reduced turnover (Yu-Ping et al., 2020). On the downside, resources spent (e.g., time and attention) to cope with inter-role conflicts are not invested in task-related activities, causing lower task performance (Methot et al., 2016; Pillemer & Rothbard, 2018). Additionally, a subordinate's overreliance on the leader's views and excessive loyalty (Boyd & Taylor, 1998) may hamper creativity and constructive feedback and even result in a greater likelihood of counterproductive or unethical pro-supervisor behaviors. Over time, role compromising may prove so exhausting that the subordinate will turn to role elimination (Van de Vliert, 1981) and ultimately leave the organization.

**P5a.** The resources generated by the blended friendship with the leader will result in (a) decreased emotional exhaustion (b) increased job engagement, (c) increased performance, and (d) decreased turnover intention.
**P5b.** The relationship between the resources related to blended friendship and performance and turnover intention will be mediated by (a) decreased emotional exhaustion and (b) increased job engagement.
**P6a.** Subordinates’ coping with inter-role conflicts related to blended friendship with the leader will result in (a) increased emotional exhaustion, (b) decreased job engagement, (c) decreased performance, and (d) increased turnover intention.
**P6b.** The relationship between coping with inter-role conflicts and performance and turnover intention will be mediated by (a) increased emotional exhaustion and (b) reduced job engagement.

### Coexisting pathways and net effect

Conflict and enrichment operate independently, such that individuals alternatively experience both (Greenhaus & Powell, 2006; Ramarajan et al., 2017). Both pathways are thus likely to coexist in each blended friendship, yet in different situations: situations characterized by a higher discrepancy between the expectations attached to each role (Ramarajan et al., 2017) may lead to inter-role conflicts while less discordant situations may foster inter-role enrichment. For example, when a subordinate shares a personal issue with the leader (i.e., activation of the friend's role) to request to leave early from work (i.e., subordinate's role), the contrast between the friendship norm of preferential treatment and the work norm of equity may become apparent (i.e., high discrepancy between roles). However, a discussion on a work task (i.e., subordinate's role) during a lunch outside work (i.e., friend's role) enables the subordinate to acquire different types of resources (e.g., advice, emotional support) within the same interaction with the leader (i.e., low discrepancy between roles).

Several factors may influence the discrepancy between roles. Structural factors such as organizational formalization, which accentuates the rigidity of work roles, may intensify dual-role tensions between workplace friends (Horan et al., 2021). Team LMX differentiation may also accentuate both inter-role enrichment and inter-role conflicts. When differentiation is high, the privileged position of a blended friend will be heightened because the leader has fewer alternative team members to distribute resources to (Anand et al., 2015), facilitating enrichment and resource gains. However, high differentiation may also engender rivalry and suspicion of favoritism (Kauppila, 2016; Pillemer & Rothbard, 2018), inciting the leader to turn down the subordinate-friend's personal requests to restore team fairness, and thus create inter-role conflicts. High team LMX differentiation may also augment the risk that the leader places excessive work demands on their subordinate-friend because other team members may not seem sufficiently trustworthy (Kauppila, 2016); the subordinate may interpret such work overload as a breach of the leader friend's role (i.e., friends are supposed to care about each other's well-being). Contextual factors, such as a subordinate's poor performance resulting in a leader's negative feedback, a high workload that the subordinate is not willing to accept, or events in the subordinate's personal life that interfere with work may also increase role discrepancy. Overall, blended friendships are subject to a mix of factors that operate in opposite directions, with some configurations being more favorable to enrichment (e.g., low organizational formalization, reasonable job demands, high subordinate performance) and others to conflicts.

**P7.** Over time, subordinates involved in a blended friendship with their leader will experience both inter-role enrichment and conflict: situations where the discrepancy between roles is minimized will foster enrichment, whereas those that emphasize the discrepancy will foster conflicts.

The overall impact of the inter-role enrichment and conflicts mechanisms could be positive, negative, or neutral if the two mechanisms offset one another. Several bodies of work suggest that the neutrality hypothesis is unlikely. The JD-R literature indicates that a combination of high resources and high demands produces increased levels of engagement (Bakker, 2014). Additionally, studies using a person-centered approach found that workers facing high levels of both conflict and enrichment (i.e., demands and resources) tend to report the highest levels of exhaustion (e.g., Rantanen et al., 2013). Thus, subordinates in a blended friendship may exhibit heightened levels of exhaustion and engagement as the resources gained through the enrichment path do not fully buffer the negative effect of coping strategies while the demands produced by coping strategies do not fully attenuate the effect of resources.

Moreover, when individuals experience work-life conflict and enrichment simultaneously, conflict affects individuals more strongly than enrichment (Huyghebaert-Zouaghi et al., 2022). Other work shows that a combination of high exhaustion and high engagement is associated with higher rates of turnover intention (Gillet et al., 2022). Therefore, we expect a “bad is stronger than good” effect (Baumeister et al., 2001), such that adverse effects induced by the inter-role conflicts path should weigh more on subordinates’ work experience than the positive effects of the inter-role enrichment path. As both mechanisms operate over time, we can minimally infer that the net effect of blended friendship should be suboptimal.

**P8.** Subordinates involved in a blended friendship with their leader will exhibit higher levels of engagement and exhaustion compared to those not involved in such relationships, resulting in weaker performance and higher turnover intention.

### Feedback loops

Since blended friendships are dynamic, feedback mechanisms are likely to operate. First, the outcomes of inter-role enrichment and conflicts (e.g., exhaustion, engagement) may exert a feedback effect on future enrichment and conflicts through the salience of the discrepancy between the friend role and the subordinate role. When the net outcome is negative, role discrepancy becomes more visible to the subordinate and leader, creating more frequent inter-role conflicts. For example, an enduring decrease in performance could prompt the leader to make unfavorable decisions regarding the subordinate's advancement and pay, highlighting the power asymmetry within the blended friendship. Similarly, an exhausted subordinate may expect extra support from their leader-friend, yet not obtain it because of organizational constraints. Conversely, when the net outcome is positive, the occurrence of such situations will be minimized.

Second, it is reasonable to expect reciprocal feedback loops from both the resources and relationship maintenance strategies to blended friendship. On the one hand, resources produced by inter-role enrichment may be reinvested in the relationship to improve its quality. This is for example likely for positive affect, which is known to foster relationship thriving (Sels et al., 2021). On the other hand, inter-role conflicts may elicit negative feelings towards the leader friend, thereby affecting the relationship. As both inter-role enrichment and conflicts occur over time, subordinates may experience a mix of positive and negative feelings (i.e., ambivalence) towards their leader-friend. While ambivalence violates individuals’ inclination for consistency (Rothman et al., 2017), most people are nevertheless reluctant to disengage from ambivalent relationships (Lee et al., 2019) because the cost of leaving is high. This may be particularly true for subordinates involved in a blended friendship because ending the relationship involves losing the resources associated with both friendship and high-LMX. Yet, subordinates may decide to terminate the relationship after a significant breach (e.g., betrayal) by the leader that transgresses subordinates’ expectations to a point that no room is left for role compromising (Dimotakis et al., 2023; Epitropaki et al., 2020). In such situations, subordinates may feel that role elimination (e.g., limiting interactions or changing teams or organizations) is the only option (Van de Vliert, 1981).

## Discussion

This paper bridges the literature on LMX and personal relationships to explain how and why high LMX relationships may shift to blended friendships and lead to positive and negative outcomes for subordinates. As such, it contributes significantly to the relational leadership and the role multiplexity literatures.

Our theorizing contributes to the relational leadership literature by positioning LMX in relation to other types of close relationships. This is a critical contribution because: (i) LMX theory has dominated research on leader-subordinate relationships in the past decades, leaving little room for other relational lenses (Thomas et al., 2013), (ii) the development of friendship-like ties is common in the workplace (Horan et al., 2021), (iii) confusion has persistently surrounded the exact nature of high LMX relationships and the potential overlap with friendships (Thomas et al., 2013), and (iv) these different relationship types may not lead to the same outcomes and failing to accurately identify the nature of a relationship can lead to erroneous conclusions about anticipated outcomes (Song et al., 2020). Our framework clarifies that high LMX and friendship are distinct relationships that, although seemingly close, differ in many respects including their structure, goals, and processes. We further argue that when friendship features emerge within a high LMX, the resulting relationship is not a high LMX relationship, a traditional friendship, or the addition of both, but rather a blended (i.e., multiplex) friendship. This clarification matters as conceptual clarity is the cornerstone of knowledge accumulation and necessitates thorough examination within nomological networks (Suddaby, 2010).

Moreover, our proposition that some high LMX relationships change over time has implications in three areas. First, we suggest that scholars should examine LMX relationships from a dynamic perspective, which is scarcely used despite wide agreement that relationships fluctuate over short and long periods (Dimotakis et al., 2023; Park et al., 2015). Recent evidence indeed indicates that routinized LMX relationships are susceptible to short-term positive or negative variations caused by mood fluctuations and incidents (Dimotakis et al., 2023; Ellis et al., 2019) as well as long-term fluctuations caused by critical events like betrayals, persistent perceptions of unfairness, or LMX differentiation (Cropanzano et al., 2017; Epitropaki et al., 2020; Park et al., 2015). Our model calls for a deeper examination of those events that not only contribute to driving higher vs. lower levels of LMX but also play a role in sparking a qualitative shift in the relationship. Second, most studies continue to treat LMX as a unidimensional construct (Dulebohn et al., 2017), overlooking the fact that LMX of equal overall quality may differ in nature (Maslyn & Uhl-Bien, 2001). Examining LMX relationships with refined information about their content—especially about their degree of personalization—would provide insights into the nature of those high LMX relationships that are more prone to transition to blended friendships. Relatedly, LMX research may have failed to detect shifts to blended friendships because current measures of LMX are not designed to do so. This is an important issue because high LMX and blended friendships may result in different outcomes. Empirical efforts to disentangle high LMX relationships and blended friendships are thus critically needed.

Our theorizing also contributes to the role multiplexity literature by enriching the understanding of the psychological processes and consequences associated with role multiplexity in leader-subordinate relationships. First, though a growing body of research has examined the outcomes of workplace friendships, the specific case of leader-subordinate blended friendships remains underexplored (Horan et al., 2021). Second, our work delves into the mechanisms (i.e., inter-role enrichment and conflicts) through which blended friendships lead to positive vs. negative outcomes for subordinates. Notably, we provide a detailed analysis of the mechanisms (e.g., resource gains, relational maintenance strategies) involved. Previous work has focused on the dialectical tensions created by closeness (e.g., Bridge & Baxter, 1992), underestimating the additional resources that role multiplexity may generate for subordinates. In line with others (e.g., Ramarajan et al., 2017), we suggest that multiplex ties require a balanced examination of their consequences. Future research may extend our work by exploring the types of expectations related to the subordinate and friend roles, and the salience of contrast between these roles, that may explain when benefits outweigh drawbacks and vice versa.

### Future research directions

Researchers undertaking empirical testing of the proposed model should address how blended friendship can be measured. Studies on multiplex workplace relationships have traditionally used the combination of instrumental and friendship ties (e.g., Methot et al., 2016), using one question to identify working relationships and another to assess friendship ties. This juxtaposition is consistent with our conceptualization of blended friendship as the overlap between high LMX and friendship. While valid measures of LMX exist, we are not aware of a scale addressing leader-subordinate friendship. Therefore, future research should develop a scale that fits the key dimensions of friendship, as examined in this paper.

Moreover, in accordance with the model's boundary conditions, an appropriate sample should be composed of subordinates involved in high LMX relationships. However, selecting such a sample requires (i) careful timing to enable researchers to observe the potential transition of LMX relationships to blended friendships and (ii) specifying a threshold for selecting high LMX relationships only (typically one standard deviation above the mean, e.g., Restubog et al., 2010). Because LMX relationships develop quickly (Nahrgang & Seo, 2015), these constraints imply that researchers should start tracking dyadic partners from the outset of their relationship and for a sufficient duration (i.e., probably at least a year; Sias & Gallagher, 2009) for a blended friendship to develop and the consequences to materialize.

Furthermore, diary studies are well suited for studying the dynamic process of social interactions (Gochmann et al., 2022), particularly the development of intimacy (Laurenceau et al., 2005) and the outcomes of leader-subordinate interactions (Martin et al., 2023). Diary studies offer granularity that helps capture momentary events (e.g., a self-disclosure, a specific role conflict) that traditional longitudinal design may not detect (Martin et al., 2023). A combination of daily or weekly assessments of self and partner disclosures, and partner responses, resources, coping strategies, exhaustion, and engagement, along with measures of blended friendship, performance, and turnover intention may be a promising option. Well-validated scales to assess the independent variables of the model exist (e.g., Kirkman et al., 2009, for power distance orientation; Kreiner, 2006, for preference for work-life integration vs. segmentation), except for personalization*.* One potential avenue could be to use the LMX-MDM scales (Liden & Maslyn, 1998) which capture both proxies of person-based (i.e., affect dimension) and role-based (i.e., contribution dimension) characteristics.

On a different note, our framework focuses on the subordinate's perception of the relationship and the effects caused by their subjective perception. However, it would be interesting to investigate the leader's perception as well. Are the consequences of blended friendships as experienced by leaders similar to those encountered by subordinates? Investigating recently promoted leaders, Unsworth et al. (2018) found that leaders experienced significant role conflicts when trying to deal with preexisting friendships, due to the power differential associated with their new role. Thus, the inter-role conflicts experienced by leaders involved in blended friendships may differ from those experienced by subordinates.

Finally, as remote work becomes the new reality for many workers (Franken et al., 2021), future research may extend and adapt our theorizing to situations where high LMX partners work remotely. Since physical proximity fosters self-disclosure (Greene et al., 2006), blended friendships may be less likely to develop in remote work settings. However, remote work increases work-life blurring (Allen et al., 2021) and opportunities arise for private life to intrude into work (e.g., a child appearing in the background), creating a favorable context for disruptive self-disclosures. Qualitative work suggests that multiplex friendships may develop among coworkers in virtual work (Schinoff et al., 2020), yet specific theorizing is needed to understand LMX relationships in virtual settings.

### Practical implications

Should our model be supported by empirical evidence, our framework would suggest that high-quality LMX relationships may gradually, yet not necessarily intentionally, shift toward closer relationships that may bring benefits but also come with challenges for subordinates. Because evidence has shown that high-LMX relationships have positive outcomes (Dulebohn et al., 2012), the conventional wisdom of keeping some distance between leaders and subordinates and some separation between work and life (Rothbard et al., 2022; Rothbard & Ollier-Malaterre, 2016) to avoid the relationship turning into a blended friendship remains valid. Studies suggest that younger professionals expect their interactions with supervisors to be open and straightforward (Pasko et al., 2021), and the growth of virtual interactions on social media and other online spaces favors more personal disclosures (Ollier-Malaterre et al., 2013). However, subordinates should be cautioned that apparent friendships and close relationships with leaders come with benefits but are not risk-free. Apparent closeness should not prevent subordinates from recognizing the power asymmetry that constrains the leader–subordinate relationship, even online (Rothbard et al., 2022). Practically, employees and leaders should be sensitized to the need to maintain some distance in their relationships on- and off-line, even if the boundary between a close and a too-close relationship is difficult to determine.

## Conclusion

This paper examines why and how high LMX may shift over time toward blended friendship and lead to a mix of positive and negative consequences. We proposed that high LMX relationships characterized by specific team, dyadic, and individual-level factors are more prone to shift toward blended friendship, entailing both inter-role enrichment and conflicts. These mechanisms generate positive and negative consequences in terms of subordinates’ well-being, engagement, performance, and turnover. We hope that clarifying the mechanisms underlying the beneficial and detrimental outcomes of these specific high LMX relationships will spark future research to disentangle the various types of leader-subordinate relationships in the workplace.
